# Mortality analysis of burns in a developing country: a CAMEROONIAN experience

**DOI:** 10.1186/s12889-020-09372-3

**Published:** 2020-08-20

**Authors:** Ndung Ako Forbinake, Claude Stephan Ohandza, Karl Njuwa Fai, Valirie Ndip Agbor, Betrand Kealebong Asonglefac, Desmond Aroke, Gerard Beyiha

**Affiliations:** 1 Meri District Hospital, Meri, Far-North Cameroon; 2 Dzeng Medicalised Health Centre, Dzeng, Cameroon; 3 Graduate School of Tropical Medicine and Global Health, Nagasaki University, Nagasaki, Japan; 4 Nuffield Department of Population Health, Oxford University, Oxford, UK; 5 St. Maria Soledad Catholic Hospital, Bamenda, Cameroon; 6 Newark Beth Israel Medical Center, Newark, NJ USA; 7 Burn Unit, Douala General Hospital, Douala, Cameroon

**Keywords:** Burns, Causes, Mortality, Cameroon

## Abstract

**Background:**

Burns are a serious public health problem worldwide accounting for an estimated 265,000 deaths annually from fires alone. The vast majority (96%) of deaths from fire-related burns occur in low- and middle-income countries and burns are one of the leading causes of disability-adjusted life-years (DALYs) in the developing world. Most burn centres are situated in large cities and are inadequate for the high incidence of injuries. An 8 year review of 440 patients in the Douala General Hospital, showed that the majority of patients burned were males (*n* = 281, 68.9%), the mean age was 25.2 ± 17.77 years with an admission rate of 69.5% (306 patients). The modal and median age were 31.0 years and 25.0 years respectively, interquartile range (0.4–82). Majority of burns (*n* = 237, 53.9%) had burn surface area ≥ 10%, most burns were 2nd degree (*n* = 215, 48.9) and the commonest burn agents were flames (*n* = 170, 37.3%), electricity (*n* = 119, 26.3%) and water (*n* = 114, 25.2%). The paucity of data on burn mortality in Cameroon motivated this study and is aimed at determining the mortality rate, causes and factors associated with death of burnt patients in the burn unit of the Douala General Hospital (DGH).

**Methods:**

It was a retrospective observational study carried out from the 1st of January 2008 to the 31st of December 2015 in the Burn Unit of the Douala General Hospital. An adapted questionnaire was used to collect demographic data, burn agents, burn depth; diagnostic delay, burn surface area, complications, comorbidity, mortality and its causes. Data was transferred to Microsoft Excel 2015 and the Statistical Package for Social Sciences (SPSS) version 20.0 for data analysis.

**Results:**

During this 8 year period, 440 patients were studied and the mortality rate was 23.4% (103 patients). The fatal burn agents were, flames (*n* = 69, 67.0%), electricity (*n* = 15, 14.6%), water (*n* = 12, 11.6%), contact (*n* = 4, 3.9%), Oil (*n* = 2, 1.9%) and chemicals (*n =* 1, 1.0%). The causes of death were shock (*n* = 36, 35.0%), sepsis (*n* = 25, 24.3%), acute respiratory distress (*n =* 25, 24.3%), acute renal failure (*n* = 6, 5.8%), severe anaemia (*n* = 4, 3.9%) and unrecorded causes (*n* = 7, 6.7%).

**Conclusion:**

A quarter of all patients died mostly from flame burns and to a lesser extent, electricity and scalds. Increase in burn depth and burn surface area worsened the prognosis. Shock (the commonest cause of death), sepsis, acute respiratory distress, acute renal failure and wound infection were significantly associated with mortality.

## Background

Burns are a serious public health problem worldwide accounting for an estimated 265,000 deaths annually from fires alone [1]. Burns are the leading causes of disability-adjusted life-years (DALYs) lost in low- and middle-income countries [1]. In 2004, about 11 million people worldwide suffered from burns, severe enough to require medical attention [2].

The vast majority (over 95%) of fire-related burns occur in low- and middle-income countries, particularly in Africa and little attention is paid to paediatric injuries [3, 4]. Burn injuries are a major cause of prolonged hospital stays, disfigurement, disability, and death in Africa [3].

Most of the advances in prevention and care have been incompletely applied in low- and middle-income countries. Increased efforts to do so would probably lead to significant reductions in rates of burn-related death and disability [1]. In order to address this public health problem, the World Health Organization (WHO) has launched *A WHO plan for burn prevention and care.* WHO would like to promote in terms of: advocacy, policy, data and measurement, research, prevention, health-care services for victims and capacity building. It includes activities that WHO itself commits to undertake in the near future as well what the entire field of burn prevention and control should be accomplishing in the coming decade [5].

Most burn centres are situated in large cities and are inadequate for the high incidence of injuries. Resuscitation is often delayed as patients have to travel long distances and transport facilities are poor. Many burn centres are also plagued with lack of resources, lack of operating time, and shortage of blood [6].

In 2014, the mortality outcome predictions in high income countries were lower than those in the DGH using the Abbreviated Burn Severity Index (ABSI) in patients admitted at the Douala General Hospital [7]. Burns and scalds are common presentations in Cameroonian health institutions but many of these patients however end with severe morbidity or even death or thrown into further poverty [5, 8]. Fire is the 20th leading condition responsible for Years of Life Lost (YLLs) in Cameroon, 107 (1%) in 2013 [9].

The paucity of data on burn mortality in Cameroon motivated this study and is aimed at determining the mortality rate, causes and factors associated with death of burnt patients in the burn unit of the Douala General Hospital (DGH).

## Methods

### Study design and setting

This study was a retrospective observational study of files in the Burn Unit of the Douala General Hospital (Cameroon) from January 1st 2008 to December 31st 2015.

This hospital is a referral hospital in the Cameroonian health system and has the only burn centre in the public sector. There are 3 hospitalisation units attributed for mild, moderate and severe burns admitted based on the American Burn Association (ABA) classification, 2 water therapy rooms and 1 operating theatre. The Unit is headed by an Anaesthetist specialised burn management assisted by a team of surgeons, anaesthesiologists, general practitioners, nurses, nutritionists, psychiatrists, nursing aids and support staff. All burn patients arriving at the DGH are ushered at the Burn Unit for immediate management. Demographic data is collected by a receptionist, history, physical examination and management prescribed by the physicians. Treatment protocol is patient dependent and the fluid management protocols usually follow Parkland Hospital’s formula, Carvajal’s Rule and Evan’s Formula. Medical treatment was through the variable use of antibiotics, analgesics, high protein and carbohydrate diet, blood transfusions, iron supplementation, stress ulcer prophylaxis etc. Surgical treatment comprised of wound debridement, skin grafting, amputations, fasciotomy, and wound care with trolamine, petroleum gel and antiseptic solutions.

### Study population

The study population was made up of burn patients treated in the burn centre between the 1st of January 2008 and the 31st of December 2015.

### Inclusion criteria

- All burned patients attended-to during the study period.

### Exclusion criteria

- All inadequate or incomplete files such as absence of age, sex, diagnosis, outcome, burn depth, burn surface area, management plan and destroyed files.
- Patients treated for Lyell’s Syndrome.

### Data collection & statistical analysis

The variables of each patient were extracted from the hospitalisation files (such as age, sex, time of arrival after injury, burn circumstance, burn surface area, burn depth, complications and outcome) then filled in the questionnaire. This information was transferred from the questionnaire into Microsoft Office Excel Worksheet 2015 and then coded into the Statistical Package for the Social Sciences (SPSS) version 20.0.The Pearson Chi-square was used to determine associations between categorical variables and mortality with the level of significance set at 0.05 (95% confidence interval).

Mortality rate was calculated thus,

$$ Mortality\ rate=\frac{\mathrm{Number}\ \mathrm{of}\ \mathrm{patients}\ \mathrm{who}\ \mathrm{died}}{\mathrm{Total}\ \mathrm{number}\ \mathrm{of}\ \mathrm{patients}}\times 100 $$

### Study limitations

Unavailability of explicit information on the presence of inhalational injuries from the case files.

## Results

During this 8 year period, 440 patients were studied, most being males (*n* = 281, 68.9%) and the admission rate was 69.5% (306 patients) [10]. The modal and median ages were 31.0 years and 25.0 years respectively, interquartile range (0.4–82). Majority of burns (*n* = 237, 53.9%) had burn surface area ≥ 10%, almost half (*n* = 215, 48.9) of all burns were 2nd degree and the commonest burn agents were flames (*n* = 170, 37.3%), electricity (*n* = 119, 26.3%) and water (*n* = 114, 25.2%) [10].

The mortality rate was calculated as follows,

$$ Mortality\ rate=\frac{103}{440}\times 100=23.4 $$

The mortality rate of this study was 23.4% (103 patients).

From this study, sex and age didn’t influence mortality. (See Tables 1 and 2).

**Table 1:** Sex distribution of patients who died

Sex	Survival (%)	Death (%)	Total (%)	***p***-value
**Male**	214 (63.7)	67 (65.1)	281 (63.9)	
**Female**	122 (36.3)	36 (34.9)	159 (36.1)	0.467
**Total**	337 (76.6)	103 (23.4)	440 (100.0)

**Table 2:** Age distribution of patients who died

Age	Survival (%)	Death (%)	Total (%)	***p***-value
**0–10**	97 (28.8)	23 (22.3)	120 (27.3)	
**11–20**	50 (14.8)	13 (12.6)	63 (14.3)	
**21–30**	64 (19.0)	21 (20.4)	85 (19.3)	
**31–40**	72 (21.4)	23 (22.3)	95 (21.6)	
**41–50**	29 (8.6)	12 (11.7)	41 (9.3)	
**51–60**	15 (4.6)	6 (5.9)	22 (5.0)	0.541
**61–70**	6 (1.8)	2 (1.9)	8 (1.8)	
**71–80**	3 (1.0)	2 (1.9)	5 (1.2)	
**> 80**	0 (0.0)	1 (1.0)	1 (0.2)	
**Total (%)**	377 (76.6)	103 (23.4)	440 (100.0)	

**Fig. 1:**
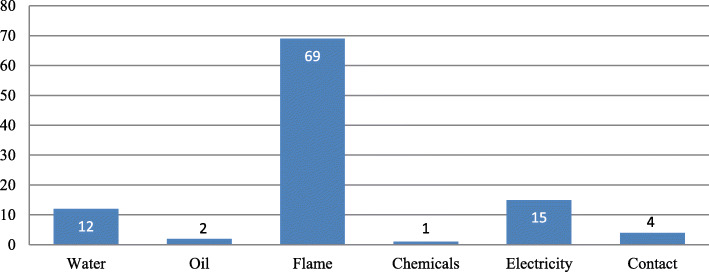
Distribution of patients who died according to burn agent

Of the 166 patients burned by flames, many (*n* = 69, 41.6%) died and generally, flame burns accounted for the majority of deaths (*n =* 69, 66.3%) in this study (See Fig. 1):

Flames, water and electricity were significantly associated with mortality as shown below (See Table 3):

**Table 3:** Burn agents and mortality

Burn Agent	Survival (%)	Death (%)	Total (%)	***p***-value
Water	94 (27.9)	12 (11.7)	106 (24.1)	**0.001**
Oil	22 (6.5)	2 (1.9)	24 (5.5)	0.031
Flame	97 (28.7)	69 (67.0)	166 (37.7)	**0.001**
Chemical	7 (2.1)	1 (0.9)	8 (1.8)	0.496
Electrical	102 (30.3)	15 (14.6)	117 (26.4)	**0.001**
Contact	15 (4.5)	4 (3.9)	19 (4.5)	0.033
Total	337 (76.6)	103 (23.4)	440 (100.0)	–

Burn depth influenced mortality as all the forms were statistically associated to mortality (*p* = 0.001). Nonetheless, it was demonstrated that having a 1st degree burn had a negative correlation with death but an increase in burn depth from 2nd to 3rd degree increased the propensity to a fatal outcome. (See Table 4).

**Table 4:** Distribution of patients who died according to burn depth

Burn deptht	Survival (%)	Death (%)	Total (%)	***p-***value	R
**First degree**	123 (36.5)	8 (7.8)	131 (29.8)	**0.001**	**−0.310**
**Second degree**	147 (43.6)	68 (66.0)	215 (48.9)	**0.001**	**+ 0.268**
**Third degree**	66 (19.9)	27 (26.2)	93 (21.3)	**0.001**	**+ 0.364**
**Total**	337 (76.6)	103 (23.4)	440 (100.0)	–	–

The total body surface area burnt was also statistically associated to mortality. (See Table 5).

**Table 5:** Mortality according to burn surface area

% Burn surface area	Survival (%)	Death (%)	Total (%)	***p***-value
**0–9**	199 (59.0)	2 (1.9)	201 (45.6)	
**10–19**	63 (18.7)	4 (2.9)	67 (15.2)	
**20–39**	55 (16.3)	24 (23.3)	79 (18.0)	
**40–59**	17 (5.1)	25 (24.3)	42 (9.6)	**> 0.001**
**≥60**	3 (0.9)	48 (46.6)	51 (11.6)	
**Total**	337 (76.6)	103 (23.4)	440 (100.0)	

The time of arrival after injury was statistically significant with respect to mortality for those who came to the burn unit between 25 to 48 h after injury (See Table 6).

**Table 6:** Mortality according to time of arrival after injury

Time	Survival (%)	Death (%)	Total (%)	***p***-value
**0–1 h**	150 (44.5)	33 (32.0)	183 (40.4)	0.005
**2–8 h**	92 (27.2)	29 (28.1)	121 (26.7)	0.040
**9–24 h**	28 (8.3)	22 (21.4)	54 (11.9)	0.758
**25–48 h**	26 (7.7)	7 (6.9)	33 (7.5)	0.001
**> 48 h**	40 (11.9)	12 (11.6)	52 (13.5)	0.733
**Total**	337 (76.6)	103 (23.4)	440 (100.0)	0.005

Many patients experienced complications during this 8 year time frame. The table below shows which of the complications were statistically significant with mortality (See Table 7). The presence of comorbidities didn’t influence mortality in this study (*p* = 0.658) (See Table 8).

**Table 7:** Mortality with respect to complications faced

Complications	Survival (%)	Death (%)	Total (%)	***p***-value
**Shock**	12 (8.3)	85	97 (223)	**0.001**
**Sepsis**	34 (23.4)	28	62 (14.3)	**0.001**
**Acute respiratory distress**	6 (4.1)	51	57 (13.1)	**0.001**
**Anaemia**	34 (23.4)	19	53 (12.2)	0.017
**Acute renal failure**	1 (0.7)	14	15 (3.4)	**0.001**
**Wound infection**	12 (8.4)	85	97 (22.3)	**0.001**
**Gangrene**	46 (31.7)	8	54 (12.4)	0.086
	145 (33.3)	290	435 (100)

**Table 8:** Mortality and presence of comorbidity

	Presence	Survival (%)	Death (%)	Total (%)	***p***-value
	Yes	6 (1.80	1 (1.0)	7 (1.6)	
**Comorbidity**					0.658
	No	33 (98.2)	102 (99.0)	444 (98.0)	
**Total**		337 (76.6)	103 (23.4)	440 (100.0	

**Fig. 2:**
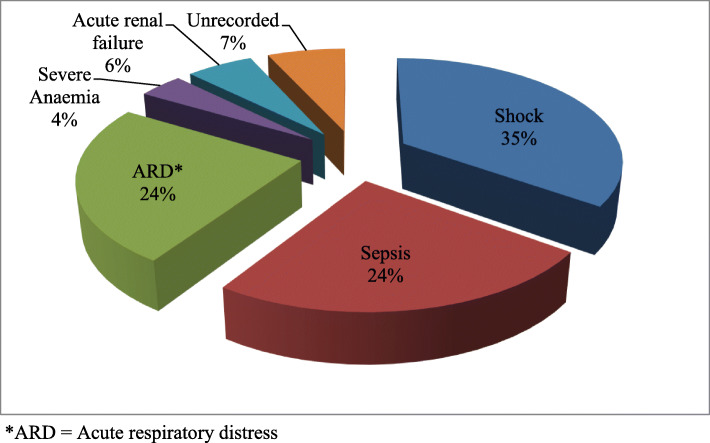
Distribution of burn mortality causes

The main causes of death were shock (*n* = 36, 35.0%) followed by sepsis (*n* = 25, 24.3%), acute respiratory distress (*n =* 25, 24.3%) and others. (See Fig. 2).

## Discussion

This paper presents an 8 year review of burn mortality in the Burn Centre of the Douala General Hospital in Cameroon. The mortality rate of this study was 23.4%. For studies which had a higher mortality rate than that of this study; according to Amengle *et.al*, the mortality was 41.2% due to the high incidence of severe burns in these children [11]. Kalayi in Zaria, Nigeria had a mortality rate of 35% was because 52% of admissions were children below the age of 5 years with severe burns [12]. Nguema *et.al*, in Gabon had a death rate of 54.8%. This was because this study was conducted in an intensive care unit [13]. Soltani *et.al*, in Tehran, Iran had a mortality rate of 59.48% because patients with below 40% of burned surface constituted 52.5% of injuries so more cases of severe burns [14].

This was similar to other studies in Cameroon, Nigeria and Morocco [15–19] which can be a reflection of the health care system in low and middle-income countries as the aforementioned countries are generally at the same stage of economic development. This study’s mortality rate was lower than other studies in Cameroon, Nigeria, Ghana and Iran [11–14] as the later compared involved more of severe burns. On the other hand, mortality rate of this study was higher than that of other studies in Nigeria, Singapore and China [20–22]. The disparity in mortality rates between the burn centre in Cameroon and others in China or Singapore; calls for urgent reforms in the Cameroonian health system via health promotion in schools, work place and improvement of fire protection systems in public places.

For studies which had a lower mortality rate than that of this study; Dongo *et.al*, conducted a study in Irrua, Nigeria in 2007 revealed a revealing a 9.72% mortality rate [21]. Song & Chua in Singapore had a mortality rate of 4.61%. This was because patients with burn size 10% TBSA and less made up the majority of admissions at 70.7% while patients with burn size 30% TBSA and more made up 8.2% [22]. Jie & Baoren in China had an overall mortality rate of 0.86%. The high survival rate, may relate primarily to the low percentages of older patients and of patients with severe burns [23].

Mortality in males 64.4% was higher than that in females 35.6%. This is similar to a study conducted by Olaitan & Jiburum in Nigeria [16]. In our study, it was found that sex and mortality were not statistically significant (*p* = 0.467). The highest mortality rate was the 0–10 and the 31–40 years age group (22.1%) followed by the 21–30 years age group (20.1%) and the lowest being those aged above 80 years; this is similar to results obtained Olaitan and Jiburum, where age and mortality were not statistically significant with *p* = 0.541. It is no secret, males are usually more active, adventurous and hence, exposed to higher risks.

From this study, the percentage survival drops as the burn surface area increases. This is because the more the surface area burned, the more fluids lost, more liability to infection and multiple organ failure. This was statistically significant in this study with *p* < 0.001.

In this study, shock (34%) was the most common cause of death, sepsis (24%), acute respiratory distress (24%), severe anaemia (4%), and acute renal failure (6%) and in 8% no specific cause of death was indicated. This is unlike a study conducted by Olaitan & Jiburum in Nigeria where acute renal failure (24 cases, 42.1%) was the commonest cause of death, septicaemia (18 cases, 31.6%), acute respiratory syndrome (5 cases, 8.7%), shock (4 cases, 7.0%), and upper gastrointestinal bleeding due to peptic ulcer and severe anaemia (1 case each, 1.8%) and in four cases (7.0%) no specific cause of death was indicated [16]. Martina & Wardhana in Indonesia, found out that death was caused by septicaemia (42.1%), multiple organ failure (31.6%), systemic inflammatory response syndrome (17,6%), and acute respiratory distress syndrome (8.7%) [23].

In this study mortality with respect to complications was statistically significant with shock, sepsis, acute respiratory distress, and acute renal failure and wound infection (all with p < 0.001) and should remain alert signs for physicians so as improve clinical care and reduce mortality.

Most patients (31.7%) who died came in within 0 to 1 h. This is because severe burns are generally rapidly rushed to the hospital, in this case, the Douala Burn Centre. This is could be due to delayed referral from primary hospital care, ignorance and poverty. Generally, referrals are a herculean task in Cameroon due to the unavailability of ambulances, lack of knowledge on availability of certain services by the patients and lack of intercommunication between health facilities and a food for thought for the ministry of public health. In this study, mortality was statistically significant with those who came 25–48 h after burn (p<0.001) this is probably because many patients were referred still unstable.

### Study strengths

This was a comprehensive 8 year overview of burn mortality in a major burn centre in Cameroon with suggestions to improve the healthcare system.

This study comes to add scientific information in a country with little data available on burn mortality.

### Study limitation

This study entailed going into hospital files, some data were not present such as the presence or not of inhalational injury and follow-up notes following discharge; relying on these, made assessment of complications and outcome following discharge not feasible.

## Conclusion

Mortality in burn patients in our setting is high as a quarter of all patients died (mostly from flame, electricity and water burns); which calls for improvements in the health care delivery sector and general sensitisation. A prospective study is also needed for an improved assessment of the effects of the different variables on burn mortality. Increase in burn depth and burn surface area reduced survival rates. Shock (the commonest cause of death), sepsis, acute respiratory distress, acute renal failure and wound infection are alert signs which physicians need to continue paying attention to in burned patients.

## Data Availability

All data analysed during this study are included in this manuscript (and its supplementary information files can be available upon request to the corresponding author).

## Abbreviation

ARD: Acute Respiratory Distress

## References

[1] World Health Organisation. Burns: WHO; 2014. Available from: https://www.who.int/en/news-room/fact-sheets/detail/burns#content [accessed Dec 14 2015].

[2] Burns: World Health Organisation, WHO; 2014. Available from: https://www.who.int/violence_injury_prevention/other_injury/burns/en/#content [accessed Dec 14 2015].

[3] Chichom-Mefire A, Fokou M. Epidemiology of paediatric injury in low income environment_ Value of hospital based data prior to the institution of a formal registration system. Afr J Paediatr Surg.html. 10.4103/0189-6725.120909 24192473

[4] Chichom-Mefire A, Mbarga-Essim NT, Monono ME, Ngowe MN. Compliance of district hospitals in the Center Region of Cameroon with WHO_IATSIC guidelines for the care of the injured_ a cross-sectional analysis. PubMed - NCBI.html. 10.1007/s00268-014-2609-9 24838483

[5] Mock C, Peck M, Peden M (2008). A WHO plan for burn prevention and care.

[6] Ahuja RB, Bhattacharya S (2004). Burns in the developing world and burn disasters. BMJ..

[7] Verla V, Beyiha G, Ebesoh DN (2014). An Assessment of Abbreviated Burn Severity Index (ABSI) in Douala, Cameroon.

[8] Olaitan PB, Iyidobi EC, Olaitan JO, Ogbonnaya IS (2004). Burns and scalds: first-aid home treatment and implications at Enugu, Nigeria.

[9] Institute for Health Metrics and Evaluation. Cameroon; 2010 [accessed Dec 15 2015] Available from: http://www.healthmetricsandevaluation.org.

[10] Forbinake NA, Dongmo G, Ohandza CS, Chichom-Mefire A, Fokam P, Beyiha G. Epidemiologic and clinical profile of burns in a tertiary hospital in sub-saharan africa. Burns Open. 2019. 10.1016/j.burnso.2019.10.001.

[11] Amengle LA, Bengono RB, Mbengono JM, Beyiha G, Minkande JZ, Abena MO. Aspects épidémiologiques et pronostiques des brulures graves chez l’enfant. Health Sci Dis. 2015;16(1) Available from: http://www.hsd-fmsb.org/index.php/hsd/article/view/474, [cited 2016 Mar 19].

[12] Kalayi GD (2006). Mortality from burns in Zaria: an experience in a developing economy. East Afr Med J.

[13] Nguema PN, Matsiegui PB, Nsafu DN (2000). Severe burns patients: epidemiology and treatment (a study of 104 Gabonese cases). Cahiers d’Études et de Recherches Francophones/Santé.

[14] Soltani K, Zand R, Mirghasemi A (1998). Epidemiology and mortality of burns in Tehran, Iran. Burns.

[15] Beyiha G, Binam F, Batamack JF, Sosso MA. Traitement et Pronostic de la brulure grave au centre des grands brulés de Douala, Cameroun [accessed Nov 03 2015]. Available from: http://www.medbc.com/annals/review/vol_13/num_3/text/vol13n3p131.htm.

[16] Olaitan PB, Jiburum BC. Analysis of burn mortality in a burns centre. Ann Burns Fire Disasters [Internet]. 2006; Available from: http://citeseerx.ist.psu.edu/viewdoc/download?doi=10.1.1.666.2474&rep=rep1&type=pdf;19(2):59. [accessed Mar 19 2016]. PMC3188036 21991024

[17] Olaitan PB, Fadiora SO, Agodirin OS (2007). Burn injuries in a young Nigerian teaching hospital. Ann Burns Fire Disasters.

[18] Datubo-Brown DD, Kejeh BM (1989). Burn injuries in Port Harcourt, Nigeria. Burns.

[19] Les Brules: Profil Epidemologique Et Elements De Prevention A Propos De 1499 Patients Hospitalises A L’unite des Brules De Casablanca, Maroc [accessed Nov 03 2015]. Available from: http://www.medbc.com/annals/review/vol_7/num_2/text/vol7n2p57.htm.

[20] Dongo AE, Irekpita EE, Oseghale LO, Ogbebor CE, Iyamu CE, Onuminya JE (2007). A five-year review of burn injuries in Irrua. BMC Health Serv Res.

[21] Song C, Chua A (2005). Epidemiology of burn injuries in Singapore from 1997 to 2003. Burns..

[22] Jie X, Baoren C (2003). Mortality rates among 5321 patients with burns admitted to a burn unit in China: 1980-1998. Burns..

[23] Martina NR, Wardhana A. Mortality analysis of adult burn patients. J Plastik Rekonstruksi [Internet]. 2013;2(2) Available from: http://www.jprjournal.com/index.php/jpr/article/view/155, [cited 2016 Mar 26].

